# Direct Nitrous Oxide Emission**s** From Tropical And Sub-Tropical Agricultural Systems - A Review And Modelling Of Emission Factors

**DOI:** 10.1038/srep44235

**Published:** 2017-03-10

**Authors:** Fabrizio Albanito, Ulrike Lebender, Thomas Cornulier, Tek B. Sapkota, Frank Brentrup, Clare Stirling, Jon Hillier

**Affiliations:** 1 Institute of Biological & Environmental Sciences, School of Biological Sciences, University of Aberdeen, Cruickshank Building, St. Machar Drive, Aberdeen AB24 3UU, UK; 2 Research Centre Hanninghof, Yara International ASA, Hanninghof 35, 48249, Duelmen, Germany; 3 International Maize and Wheat Improvement Center (CIMMYT), Sustainable Intensification Program, NASC complex, New Delhi 110012, India; 4 International Maize and Wheat Improvement Center (CIMMYT), Sustainable Intensification Program, Apdo. Postal 6-641 06600 Mexico, D.F., Mexico

## Abstract

There has been much debate about the uncertainties associated with the estimation of direct and indirect agricultural nitrous oxide (N_2_O) emissions in developing countries and in particular from tropical regions. In this study, we report an up-to-date review of the information published in peer-review journals on direct N_2_O emissions from agricultural systems in tropical and sub-tropical regions. We statistically analyze net-N_2_O-N emissions to estimate tropic-specific annual N_2_O emission factors (N_2_O-*EFs*) using a Generalized Additive Mixed Model (GAMM) which allowed the effects of multiple covariates to be modelled as linear or smooth non-linear continuous functions. Overall the mean N_2_O-*EF* was 1.2% for the tropics and sub-tropics, thus within the uncertainty range of IPCC-*EF*. On a regional basis, mean N_2_O-*EFs* were 1.4% for Africa, 1.1%, for Asia, 0.9% for Australia and 1.3% for Central & South America. Our annual N_2_O-*EFs*, estimated for a range of fertiliser rates using the available data, do not support recent studies hypothesising non-linear increase N_2_O-*EFs* as a function of applied N. Our findings highlight that in reporting annual N_2_O emissions and estimating N_2_O-*EFs*, particular attention should be paid in modelling the effect of study length on response of N_2_O.

In the past century, to meet the increasing demand for food from a growing global population, agriculture has undergone a global “green revolution” that introduced high yielding cereal varieties together with improved agronomy including use of mineral fertilizer. Since the 1950 s, global use of mineral fertilizers has increased from 4 to 120 ± 10% TgN yr^−1^, and countries in non-OECD (Organisation for Economic Co-operation and Development) Asia, Latin America and Africa have become the main consumers with approximately 70% of the total reactive nitrogen (N_r_) produced^1^. Increasing use of excess nitrogen (N) fertilizers at low nitrogen use efficiency (NUE) is the primary driver for increased Nr losses like emission of nitrous oxide (N_2_O), volatilization of ammonia (NH_3_), leaching of nitrate, and other reactive nitrogen forms resulting in the perturbations of the global nitrogen cycle^2^,^3^,^4^. In consequence, in the past decade, the unintended N losses from the agricultural soils have discharged around 39–95 Tg N yr^−1^ in fresh waters, 30–40 Tg N yr^−1^ as NO_x_ in the atmosphere, and about 40-66 Tg N yr^−1^ to coastal waters^5^,^6^,^7^,^8^.

At the global scale, the increase of the agricultural N inputs has been mostly coupled with a decrease in nitrogen use efficiency (NUE), resulting in only 20–30% of the N inputs in agriculture being converted into food for human consumption^8^. The decline of global NUE in agriculture has been primarily linked to the imbalanced nutrient supply i.e. the application of too much nitrogen fertilizer in some regions and too little in others^9^. In the developing countries of sub-Saharan Africa and Latin America sub-optimal fertilization, coupled with inappropriate management of agricultural soils, has resulted in a loss of soil carbon (C) and associated nutrients leading to low crop yields and low nutrient content of harvested products^10^. While, in non-OECD Asian developing countries heavy subsidies on fertilizers have resulted in increased fertilizer input and substantial pollution.

The perturbation in agricultural N_r_ inputs and the associated increase in N_2_O emission has been the subject of international meetings within the United Nations Framework Convention on Climate Change (UNFCCC). More accurate greenhouse gas (GHG) emission factors (*EF*) across agricultural systems are urgently needed from developing countries to improve current and future estimations of global agricultural N_2_O emissions^11^,^12^. Current reporting of agricultural N_2_O emissions by developing countries is still largely based on the use of Tier 1 methods developed in 2006 by the Intergovernmental Panel on Climate Change (IPCC) and mostly based on data from temperate countries^13^,^14^. The under-representation of data from tropical and sub-tropical countries raises concerns about the relevance of the IPCC’s N_2_O*-EF* in countries or regions having climatic conditions and agricultural management different than the global mean^12^,^15^,^16^. A growing number of studies have highlighted that N_2_O emissions are a function of N input rates, climate, soil and the chemical form of nitrogen applied (fertilizer type)^17^,^18^,^19^. Several authors also indicate that N_2_O emissions increase nonlinearly with increasing N application rate (Shcherbak *et al*., 2014) - contradicting the assumption of linearity in the 1% *EF* (IPCC, 2006). This means that in under-fertilized or over-fertilized croplands the application of IPCC-*EF* would result in an over- and under-estimation of N_2_O emissions by 25 and 20%, respectively^20^,^21^.

At present, only a few developed countries (countries listed in Annex 1 of the UNFCCC) have implemented national and sub-national approaches to obtain more representative and spatially disaggregated N_2_O-*EFs*, and up to 93% of the developing countries still use Tier 1 methods for estimating N_2_O emissions from the agricultural sector^22^, which in particular suppose that 1% of applied N is emitted as N_2_O (direct nitrous oxide emissions from fertilizer use in field). In order to assess the adequacy of the IPCC-*EF* in developing countries more specific *EFs* need to be produced^15^. Here, using up-to-date peer-reviewed research evidence we conducted a revision of fertilizer-induced N_2_O emissions across tropical and subtropical regions of the world (Fig. 1). To this end we reviewed studies that reported N_2_O emissions in agricultural fields as affected by fertilizer application compared with an unfertilized control. We compared the fertilization effect across different N inputs, fertilizer types, mode of application, and site-specific edaphic and climatic conditions. Since net-N_2_O-N emissions may depend on multiple factors in an additive, non-linear or interactive manner we applied a Generalized Additive Mixed Model (GAMM) to assess the effects of multiple potential explanatory variables on N_2_O-*EF*, including continuous functions of N application rate and experiment duration. This modelling approach provides a statistical exploration of published data that can be used to guide further studies aimed at improving our knowledge of the factors influencing the relationship between N_2_O-*EF* and N input in the tropics.

## Results

### Tropical and sub-tropical net-N_2_O-N emissions

Across the complete dataset for the tropics and sub-tropics (Table S1) mean net-N_2_O-N emissions were 1.4 ± 3.3 kg N ha^−1^ (*n* = 247). Central & S. America had the highest fertilizer-induced net emissions (3 ± 5.1 kg N ha^−1^), followed by Australia > Asia > Africa. Africa had the lowest mean net-N_2_O emissions, while studies from Central & S. America and Asia showed the widest range of net emissions (Fig. 2). Due to high emissions under Oil palm and Sugarcane cultivation, the perennial crops (*PC*) studies showed net-N_2_O-N emissions close to 6- and 5-fold higher than those in annual crops including non-flooded rice (*AC*) and flooded rice (*R*), respectively.

Across the 42 studies selected in the statistical analysis, *Study length* ranged from 15 to 840 days with a mean 159 ± 127 days across 247 values. The dataset from Australia corresponded to a mean experimental length of 363 ± 7 days (*n* *=* 10), while the dataset from Central & S. America, Asia, and Africa corresponded to 181 ± 203 (*n = *61), 150 ± 81 (*n = *127), and 115 ± 42 days (*n* = 49), respectively. The net-N_2_O-N increased with *Study length*, and was 1 ± 1 kg N ha^−1^ for experiments up to 30 days (*n* = 20), 1 ± 3 kg N ha^−1^ for experiments above one month and up to 180 days (*n* = 168), and 3 ± 5 kg N ha^−1^ for experiments above 180 days (*n* = 59). Mean net-N_2_O-N from only the studies of length approximately 1 year (between 348 and 382 days) was 3 ± 5 kg N ha^−1^ (*n = *20), and approximately 52% higher than the mean cumulative net-N_2_O-N of the whole dataset.

### Statistical modelling of net-N_2_O-N emissions

N_2_O emission data were cube root transformed prior to fitting models and assumed to follow a normal distribution. From the 19 factors initially involved in the model selection (Table 1) only the five *viz. Study length, Crop type, Soil texture, N rate*, and *Fertilizer type* had a statistically significant effect on net-N_2_O-N emissions (Table S2). Overall, the best candidate model reported in Equation 3 had an adjusted R-squared (R^2^-adj in Table S.2) of 0.57. N fertilization was the best predictor of net-N_2_O-N emissions with the main positive and fixed effect of N rate being highly significant. Within the fertilization treatments, however, the effect of the rate of N fertilization on N_2_O emissions depended on the type of fertilizer, as shown by a significant interaction between *Fertilizer type* and *N rate*. During the model selection, described in the Supplementary information, the effect of N rate on N_2_O emissions was tested for potential non-linear trends across the dataset using smoothing splines. Ultimately the allowance for non-linearity in the effect of N rate did not improve the predictive power of the model, and the effect of N rate resulted to be best supported as fixed (linear) term.

In particular, the response of net-N_2_O-N emissions for the 8 groups of fertilizer type showed that only Ammonium Nitrate (*AN*) and Urea with Nitrification inhibitor (*U&NI*) treatments were distinguishable from the other 6 fertilizer types (Fig. S1a). On this basis the parameter *Fertilizer type* was further aggregated into three major groups: *AN, other N-fertilizers*, and *U&NI* (Fig. S1b). This procedure did improve the overall parsimony of the GAMM model reported in Equation 3, lowering the AIC value during the model selection and enhancing the significance of the interaction between *Fertilizer type* and *N rate*. The predicted net-N_2_O-N responses increased more than proportionally to increasing rates of *AN* and *other N-fertilizer* classes. In the case with N-inhibitor added to Urea the net emissions were linear and the slope close to zero (Fig. S2).

*Crop type* was the only factor directly linked to the experimental length of the studies showing a significant positive fixed effect in the model (*p*-value = 0.048). When partitioned among three broad groups of crop type, *PC* and *R* showed a greater effect than *AC* on net-N_2_O-N emissions (Fig. S3, Table S2). Finally, the smoothed function of *Study length* by *Crop type* in the GAMM model (Eq. 3) permitted to significantly improve model fit by modelling net-N_2_O-N over the length of the study (Table S2).

### Modelled net-N_2_O emission and N_2_O-EF

The overall modelled net-N_2_O-N emission across the tropical dataset ranged from −3 × 10^−0.3^ to 25 kg N ha^−1^ (mean 1.2 kg N ha^−1^), with mean net-N_2_O-N emission from Central & S. America > Australia > Africa ≈ Asia (Table 2). For *PC* the modelled emissions were on average approximately 79% and 72% higher than in *AC* and *R,* respectively. Annual net-N_2_O-N emissions were calculated by calculating predicted values from the model, setting *Study length* to 365 days. As the subpopulation corresponding to *R* was characterized by only studies with length shorter than six months, the prediction for flooded rice (17.8 kg N ha^−1^ yr^−1^, C.I. of 1.6‒70) was considered to be inaccurate at an annual basis and excluded in the analysis of the annual net-N_2_O-N emissions and subsequent N_2_O-*EF* in tropics. Overall the modelled mean annual net-N_2_O-N emission in tropics was 2 kg N ha^−1^ yr^−1^ (0.1‒22), which is on average 57% lower than the mean observed net-N_2_O-N values (Table S1) and corresponded to studies of approximately 1 year in length (3 ± 7 kg N ha^−1^, *n = *20). The mean (modelled) annual N2O-*EF* was 1.2 (0.1-7.8%) across the whole dataset, 0.9% (0.2‒3) for Australia, 1.1% (0.1‒8) for Asia, 1.3% (0.1‒7) for Central & S. America, and 1.4% (0.3‒5) for Africa (Table 2). Modelled annual net-N_2_O-N emission increased more than proportional with the N application rates (Fig. 3a). Whereas, across studies with different experimental length it did not show any specific pattern (Fig. 3b). The annual N_2_O-*EF* was 2.1% (0.6‒7) in croplands fertilized with *AN*, 1.1% (0.2‒8) in croplands fertilized with *other N-fertilizers,* and 0.7% (0.1‒3) with *U&NI* (Table 2). Mean N_2_O-*EF* decrease with the N application rates approaching the 1% in crops fertilized above 300 kg N ha^−1^ (Fig. 3c). While, in studies longer than six months the N_2_O-*EF* decreased below the 1% (Fig. 3d).

## Discussion

The underrepresentation of tropical and sub-tropical agricultural soils in global N_2_O emissions studies represents a major limitation in the development of accurate N_2_O-*EF* for these regions. The rising demand for food is placing greater pressure on land and increasing the intensification of agriculture^9^,^23^, with the result that agricultural GHG emissions are projected to increase in developing countries in the coming years^24^.

To our knowledge this is the first study reporting a review of agricultural N_2_O-*EFs* measured exclusively across the tropical and sub-tropical regions. To date, several studies discussed the consequences of using non-linear (i.e., exponential) rather than linear models (i.e., IPCC 1%-*EF*) to assess agricultural N_2_O emissions^18^,^20^,^21^,^25^. A common understanding from these studies is that the use of the IPCC-*EF* could lead to inaccurate regional estimates if the true response of the N_2_O emissions to N-fertilizer is non-linear. Shcherbak *et al*.^20^ reported that compared to their non-linear models the IPCC-*EF* would underestimate and overestimate N_2_O emissions in croplands fertilized above and below the threshold of approximately 150 kgN ha^−1^, respectively^20^. The projection shown in that study, however, is an average trend of their N_2_O-*EF* responses developed using studies with a range of lengths. In reviewing and reanalysing that data we observed that the discrepancies between the 1% IPCC-*EF* and their modelled N_2_O-*EF* would diminish when the length of the studies approach 365 days (data not shown). The 1% *EF* from IPCC is based on N_2_O emissions projections annualized using a linear mixed-effect model with a constant term for measurements covering a period of > 300 days^26^. Our results, where we estimated the effect of study length on N_2_O emissions using a smooth function distinct by crop type do not support this hypothesis that N_2_O emissions are significantly different from the linear 1% IPCC-*EF*. Indeed, our model suggested that below a fertilization of 200 kg N ha^−1^ the IPCC-*EF* would tend to underestimate N_2_O emissions by approximately 21% on average, compared to the predicted annual N_2_O-*EF* (Fig. 3c). However given the limited nature of this dataset and the multiple factors potentially affecting N_2_O emissions we strongly advise that further data is essential before robust claims of this effect could be made. Overall, the tropical mean N_2_O-*EF* was 0.2% higher than the IPCC-*EF* with a 95% confidence interval ranging from 0.1% to 8%; thus 46% greater than the uncertainty range of the global IPCC-*EF* (0.3-3%). From the study of Bouwman *et al*.^2^ which reported disaggregated N_2_O-*EFs* for continental regions the N_2_O-*EF* in West, East and Southern Africa was 5.1% and 3.1%, in Latin America 3.3% and 2.5%, and in Southern Asia and Oceania regions 3.4% and 2.5%, in arable land and grassland respectively. While, Gerber *et al*.^21^, reported mean N_2_O-*EFs* of 0.6% and 0.8% in sub-Saharan Africa and in India, respectively. Our modelling study showed that in Africa N_2_O-*EFs* would range from 0.3 to 5.3% (mean, 1.4%), in Central & South America from 0.1 to 6.7% (mean, 1.3%), in South Asia from 0.1 to 7.8% (mean, 1.1%), and in Australia from 0.2 to 2.6% (mean, 0.9%). The tropical N_2_O-*EF* derived from studies based on annual crops was on average 6.5% higher than studies carried out in perennial crops. While studies based in perennial croplands showed a wider C.I. range of N_2_O-*EFs* than annual crops.

### Progress and limitations of the literature on N_2_O emissions

In the past decade the national research capacity across tropical and sub-tropical Asian countries has improved producing positive trends in the number of publications in peer-reviewed journal^27^. To the best of our knowledge and considering only the agricultural studies reporting on agricultural N_2_O emissions from fertilized and non-fertilizer plots, 38 new studies were included in the present review of tropical N_2_O studies in addition to 8 studies considered in the last global review of 2006^28^. In spite of this literature increase, there are still major limitations in terms of spatial coverage and missing information describing soil N_2_O emissions. In addition, due to inconsistencies in reporting, we could not include parameters related to management practices such as: irrigation, tillage, liming, and crop residue treatments, and parameters relating to crop production such as: harvested biomass yield, dry matter, and crop N-uptake (Fig. S4) in the analysis. The information on soil type, soil C, and soil N remained limited due to differences in nomenclature and analytical methods across the studies. Furthermore, information on N inputs from atmospheric N deposition and plant N fixation is still missing from the majority of studies.

### Modelled annual N_2_O emission factors

Our non-linear mixing model included also N_2_O values below zero, Nitrification Inhibitors (NI) or other additives (e.g. Urease Inhibitors, coatings, etc.), and for the first time modelled the information of studies of any experimental length. In general the N_2_O emissions measured in the field tend to increase proportionally with the length of the experiments. Annual studies, in particular, have the advantage of averaging on an annual basis the impact of a complete cropping season and the related management practices. On the other hand, short-term studies can permit more accurate insight into distinct treatment effects, helping to identify emission hotspots and best management practice. By including studies of all lengths, the smoothing spline allowed for both the advantages and disadvantages of long- and short-term studies in the final annual outputs, averaging potential high and low N_2_O emissions due to wet and dry tropical seasons and intrinsic experimental differences^29^,^30^. The net N_2_O-N emissions from our GAMM model did not show any pattern of variation across studies of different length (Fig. 3b). In contrast, the annual N_2_O-*EF* declined with *Study length* (Fig. 3d). Compared to the IPCC-*EF* the median predicted N_2_O*-EF* ranges from + 18% to −18% for studies shorter and longer than 6 months, respectively. This negative relationship between our predicted *EFs* and the length of the studies reflects the rounding effects of the smoothing spline approach described above. In addition, the highly skewed residuals of the tropical dataset and the conservative statistical approach used here reduced the number of parameters used in the GAMM model. The total N rates applied during the experiments represented the most significant driving factor of N_2_O emissions. While, the effect of fertilizer type was only significant when analysed in interaction with Nitrogen rates applied. One possible explanation could be that only very few studies actually compared different fertilizer types at different N application rates within the same experiment. As a consequence, it is not possible with this dataset to derive N rate-independent N_2_O-*EFs* for single fertilizer types. Furthermore, nitrogen fertilizer types (i.e. nitrate, ammonium, urea, and mixtures thereof) behave differently in terms of N_2_O emissions depending on soil and climatic conditions. The most important factors are soil moisture, soil carbon content, and soil pH^31^,^32^,^33^,^34^. For example, nitrate-containing fertilizers such as AN tend to release more N_2_O if denitrification is supported by wet and therefore partly anaerobic soil conditions accompanied by high soluble soil carbon content^35^,^36^,^37^. Whereas urea and ammonium fertilizers can release higher rates of N_2_O under rather dry conditions^38^,^39^. Thus, as previous regional and global empirical analysis on literature datasets the present study was unable to proper consider these factors in the model.

### Statistical modelling of N_2_O emissions in agricultural systems

To date, several empirical methods have been applied to analyze agricultural N_2_O emissions. These could be summarized as: fixed regression parameter models^40^, multiple linear regression models^20^,^41^, and a number of different linear mixed-effects models^2^,^21^,^25^,^28^. Depending on the spatial and temporal scale of the dataset, empirical models on agricultural N_2_O emissions were reported to be valuable tools to estimate annual emissions at the global scale^42^, or an inaccurate and even unsuitable approach at finer spatial and temporal scales^43^. Frequently the reasons for these contrasting research outcomes lie in the quality of the datasets and the statistical approaches used to develop the empirical models.

Statistical methods such as linear mixed-effect models (LMMs), and successive extensions, have become widely used to overcome some of the limitations described above in the dataset. These models, in particular, through the use of regression parameters and variance parameters permit a variety of correlated patterns in the data to be modelled. The complex nature of LMMs, however, still represents a challenging problem in the variable selections and parameter estimations^44^. Philibert *et al*.^25^, modelling the dataset reported in Stehfest and Bouwman^28^, explored how the number and the specification of fixed- and random-effect factors can potentially affect the outcomes and interpretability of LMMs. In that respect, it is important that both random intercepts and random slopes are carefully considered in the model selection of LMMs to guard against anti-conservative conclusions (i.e., accepting an experimental effect as significant more frequently than is warranted by the data)^45^.

Given the concerns described above, and that direct N_2_O emissions from agriculture depend on a multitude of complex and sometimes unknown factors, here we used for the first time GAMM to statistically analyze the N_2_O emissions in tropical agricultural systems. GAMM is frequently applied in over-dispersed and correlated data^46^, to provide a more flexible procedure in order to describe the variability across the response and attribution of this variability to different parameters. Here, rather than specifying the nature of the relationship between agricultural factors and N_2_O responses, we used a data-driven statistical approach which permitted the modelling of the nonlinear responses of net-N_2_O emission in the dataset. The semi-parametric mixed model reported in Eq. 3 included two smoothing splines (ƒ_1_, ƒ_2_); the first to estimate the nonlinear effects of the covariate *Study length* and the second as an alternative way of specifying random coefficients for each level of the categorical factor *Crop type*, respectively. In addition, in the spirit of maximizing the random-effects structure^47^, the model included also nested random effects hierarchically structured as: “Experimental IDs” within “Study IDs” within “Countries”, which lead to a parsimonious models with a smaller than theoretically possible number of model parameters.

In the GAMM, however, *Study length* was considered as a continuous variable and its effect was modelled including the outcomes of studies ranging from few weeks to more than one year of length. Compared with the classical categorical approach, the non-parametric procedure avoided possible biases resulting from assuming a common mean *Study length* effect across distinct subpopulations of studies. An important aspect of estimating N_2_O emissions on an annual basis is the possibility of incorporating both short and long-term studies, possibly covering the whole calendar year. In the subpopulation corresponding to flooded rice, for instance, the relatively short experimental length of the studies (i.e., less than six months) resulted in very high estimates of N_2_O emission on an annual basis. Given the above limitation, flooded rice was omitted from the final stage of the analysis to estimate annual N_2_O emissions and successive N_2_O-*EF*.

## Methods

### Literature trawl and collection of data

For the collection of data on direct N_2_O emissions from arable cropping systems, we extracted all studies including N_2_O measurements in arable fields from the Stehfest and Bouwman^28^ dataset and extended this dataset by studies published between 2004 and 2016.

In order to derive the final dataset for the tropics and sub-tropics (as published in Table S1) we have searched the scientific literature according to the following steps.

**Figure 1:**
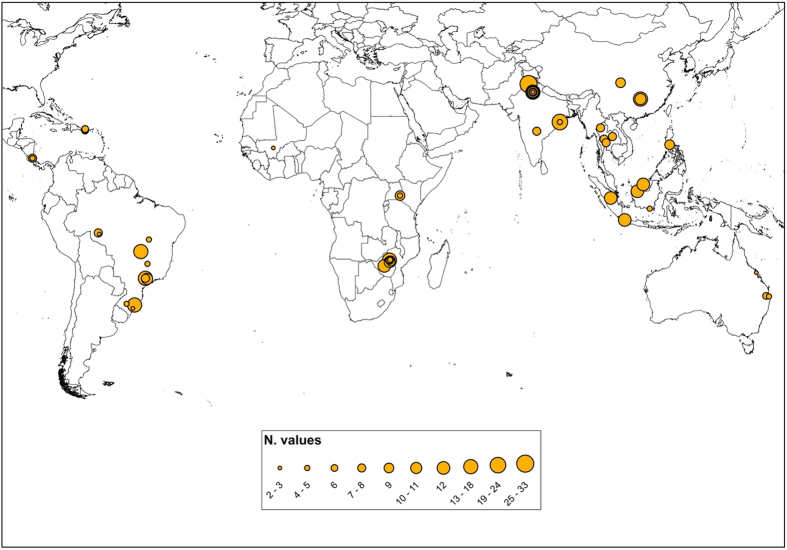
Distribution of the study used in the analysis across the tropical (23.5° North and 23.5° South latitude) and the sub-tropical (30° North and 30° South latitude) regions. Studies are reported using graduate symbols classes corresponding to the number of N_2_O values (N. values) reported in each study. Map generated using ESRI 2011. ArcGIS Desktop: Release 10.1. Redlands, CA: Environmental Systems Research Institute.

**Figure 2:**
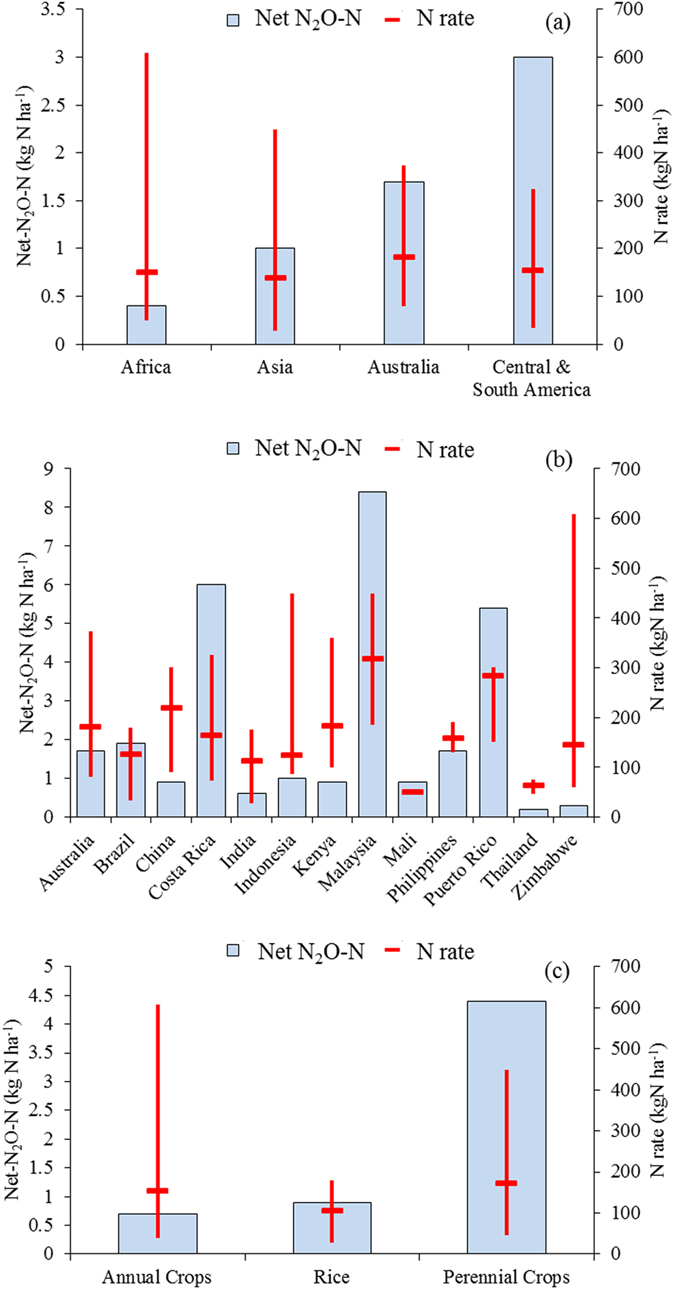
Summary of published data on net-N_2_O-N (**a**), and N rate (**b**) from studies in tropical and sub-tropical regions of the world distinct among continental regions, countries, and crop types. The red lines extending vertically in the boxes highlight the mean, minimum and maximum value of N rates.

**Figure 3:**
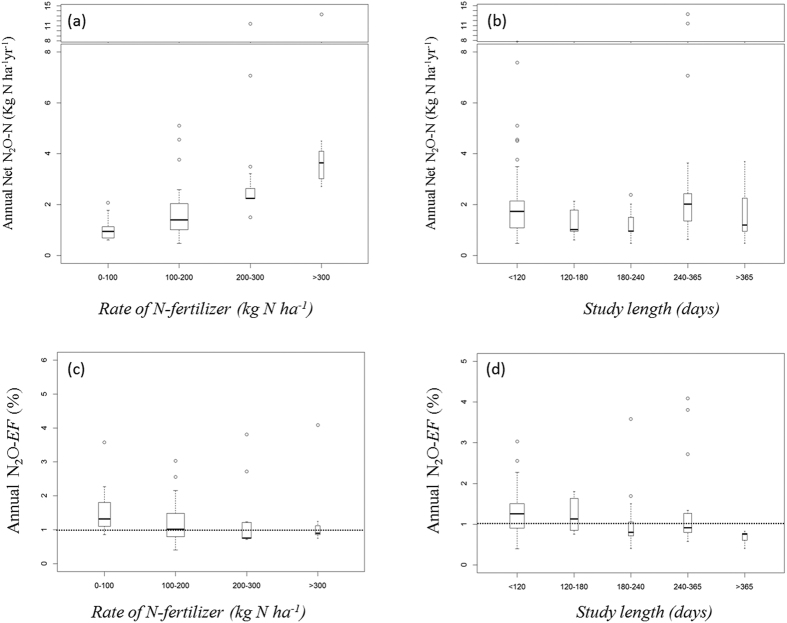
Modelled annual net-N_2_O-N and N_2_O-*EF* across distinct N rates (**a,c**) and study lengths (**b,d**) estimated using the GAMM model (Eq. 3) and the tropical dataset reported in Table S1. To estimate net-N_2_O-N on an annual basis the smoothed factor *Study length* was set at 365 days. Top and bottom of the boxes are the first and third quartiles, and the band inside the box is the second quartile (median). Sample size (*n*) differences are reported by scaling the box plot width in proportion to √*n*. Lines extending vertically from the boxes show the extreme of the lower quartiles. Outliers are plotted as individual points. The horizontal dashed lines highlight the 1% IPCC-*EF*.

1. ISI-Web of Knowledge, Google Scholar and Scopus were searched for the keywords “nitrous oxide”, “N_2_O” in combination with “fertilizer”, “Nitrogen” and “fertilizer use”. This search was complemented with a search through the literature cited in several peer reviewed review articles. In total 1144 publications were found.
2. Only data from original studies measuring direct N_2_O emissions from arable fields were included (637 publications) but no data from process modelling, lab and greenhouse experiments or review articles were considered.
3. From the remaining papers only data carried out in the tropical region (between latitudes 23.5° North and 23.5° South) and the sub-tropical ridge (between latitudes 30° North and 30° South) of the globe were considered (Fig. 1).
4. As a next step only measurements from arable fields with fertilized treatments as well as a non-fertilized control (background emission) were considered.
5. Additional mandatory parameters for papers to be included in the final datasheet were information on total nitrogen applied and the cumulative N_2_O emissions over the experimental period. Both parameters are needed to calculate the fertilizer-induced N_2_O emission factor. This resulted in 38 papers included in the present review regarding direct N_2_O emissions from tropical crop production systems.
6. Cumulative N_2_O values were extracted either directly from tables or text, or were derived from graphs using PlotDigitizer software (https://sourceforge.net/).
7. The same selection process was also applied to the Stehfest and Bouwman^28^ dataset, which resulted in 8 additional publications.

In total, 46 studies comprising 360 N_2_O measurements fulfilled the quality criteria described above were used for this statistical analysis (Table S1).

### Dataset summary

The tropical dataset contains a broad range of soil properties, fertilizer properties, cultivation practices, and crop types. Therefore, to reduce heterogeneity across the Table S1, some parameters were aggregated into fewer classes. The soil types reported in the studies were classified using the United State Department of Agriculture (USDA) soil texture classification systems which is based on the sand, silt and clay soil content. This permitted classification of approximately 74% of the 360 study values into 9 soil texture classes. The initial 24 crop types (*Crop type*) reported in the studies were aggregated into 4 broad categories: flooded rice (*R*), annual crops including non-flooded rice (*AC*), perennial crops (*PC*), and bare soil (*BS*). The fertilizer types (*Fertilizer type*), or combination of fertilizers applied in the field, where classified into 12 categories based on their primary N composition : Ammonium Nitrate (*AN*), Ammonium (*A*), Ammonium with Nitrification inhibitor (*A&NI*), Urea (*U*), Urea with Nitrification inhibitor (*U&NI*), Urea with additives (*U&AD*), Potassium, Sodium and Calcium Nitrate (*Ni*), Nitrates with Nitrification inhibitor (*Ni&NI*), mixture of Mineral-N (*Min-N mix*), Organic mix with Mineral-N (*O-Min-N mix*), Organic (*O*), and biological N fixing crops (*BNF*). The different modes of application (*Appl. Mode*) described in the studies were grouped into four categories: broadcast, incorporated, sub-surface banding, and mixed application (see Table 1). Where climatic data were not reported in the studies these gaps were filled from different sources: long term precipitation (*Prec*) was estimated using the CHIRPS database^48^. Whereas, long term temperature (*Temp*) values were found from the open climate database of Berkeley Earth (http://berkeleyearth.org/data/).

### Statistical modelling of the main drivers of net-N_2_O-N emissions

Given the absence of some reported variables in some instances in Table S1, several key parameters could not be included in the statistical and modelling analysis. These are categorical parameters such as: cation exchange capacity (CEC), soil drainage (drainage), and the continuous variables related to crop production such as: dry matter, biomass yield and crop N uptake. Due to heterogeneity in the methodologies used and uncertainties in the estimation obtained from the studies, we excluded estimates of total soil C and N content, as well as information on crop planted before the experimental period (pre-crop). N_2_O measurements from 4 fertilizer groups (*Ni, Ni&NI, BNF, A&NI*) were not included in the statistical analysis due to their insufficient representation (4 values for *Ni*, 4 values for *BNF*, 2 values for *A&NI* and 1 value for *Ni&NI*). The study of Mazzetto *et al*. 2014 based in Brazil was excluded due to the unusual high level of organic N applied as manure (1300 kgN ha^−1^). In addition, we excluded the four N_2_O measurements based on *BS*.

Net-N_2_O-N emission (kgN ha^−1^) was calculated across the whole dataset as the difference between the N_2_O emissions in a given fertilizer treatment and its respective zero-fertilizer control:


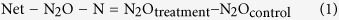


If a study applied multiple N rates for each fertilizer treatment, we calculated the net-N_2_O-N emission using its common zero—fertilizer control. While, the N_2_O-*EF* was estimated using the following equation:


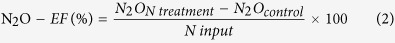


which refers to the proportion of fertilizer N that is directly released as N_2_O-N during the measurement period after discounting background emissions (i.e. emissions from unfertilized control plots).

**Figure 4:**
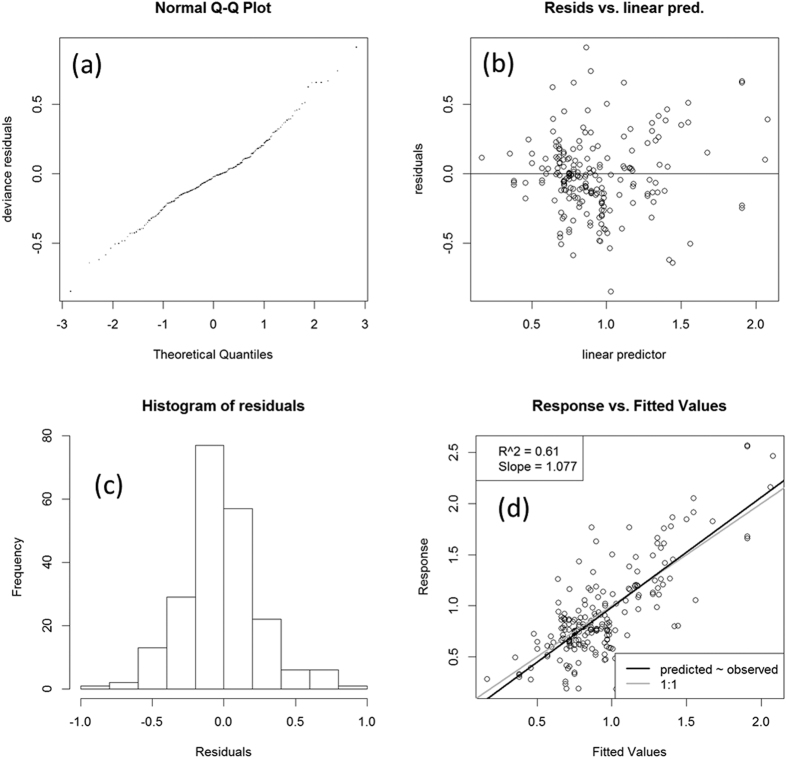
Diagnostic plots of the GAMM model reported in Eq. 3. The model assumed Gaussian family distribution for the response and equal variance for all the observations. The normal Q-Q graph (**a**) is very close to a straight line, suggesting that the distributional Gaussian assumption was reasonable for the net-N_2_O-N transformed to the cube root. The residual versus fitted values (linear predictor) (**b**) suggests that variance was approximately constant as the mean increased. The histogram of residuals (**c**) appeared approximately consistent with normality. Graph (**d**) of responses against fitted values showed a correlation of approximately 0.61.

The tropical dataset was initially described using univariate and bivariate correlation between variables (Fig. S5). To gain insight into the main drivers of net-N_2_O-N emission in tropical croplands, 19 factors across the dataset were examined using Generalized Additive Mixed Model (GAMM)^46^ (see Table 1). The statistical analysis in GAMM allows the impact of Study length on cumulative net-N_2_O-N to be modelled as a smooth non-linear function modified by Soil texture. The approach to determining the best candidate statistical model with graphical is described in the Supplementary information.Figure 4 shows a summary of the standard validation graphs to verify the normality, homogeneity, independence and overall fitness of the model. While a description of the model-term effects is reported in the Supplementary information Table S2 and Fig. S1, S2, and S3.

The best candidate semi-parametric additive mixing model was:


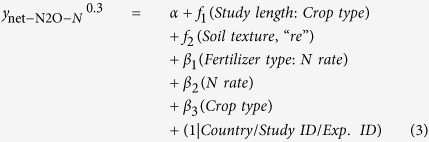


This modelled the trend of net-N_2_O-N emission (*y*_net-N2O-N_^0.3^), transformed by taking the cube root due to the highly skewed residuals, over the study length (*Study length*) distinguished between ANFC, FC, and PC crop types (*Crop type*). While the factors *Crop type, N rate* and *Fertilizer type* in interaction with corresponding *N rate* were included as fixed-effect parameters. The effects of *Study length* were modelled using the non-parametric penalized thin plate regression splines (ƒ_i_). In order to allow crossed, as well as nested random effects, intercepts across *Soil texture* were specified using the conventional random effect in GAMM using the smoothed random effects ƒ(…, bs = ”*re”*), while other random effects across the dataset were specified using hierarchically nested levels of experiment identity within study identity within study location (*Country/Study ID/Exp. ID*). The 95% confidence interval (C.I.) of the predictions from the model was used to define lower and upper limits, corresponding to the best-case and worst-case N_2_O emission scenarios, respectively. GAMMs were implemented in the R software using the packages “mgcv” and “nlme”^49^,^50^,^51^,^52^.

## Additional Information

**How to cite this article:** Albanito, F. *et al*. Direct Nitrous Oxide Emissions From Tropical And Sub-Tropical Agricultural Systems - A Review And Modelling Of Emission Factors. *Sci. Rep.* **7**, 44235; doi: 10.1038/srep44235 (2017).

**Publisher's note:** Springer Nature remains neutral with regard to jurisdictional claims in published maps and institutional affiliations.

## Supplementary Material

Supplementary Information

Supplementary Dataset 1

Supplementary Dataset 2

## Figures and Tables

**Table 1:** Parameters reported in Table S1 and used in the statistical analysis.

Parameters used in the model selection		Model terms
Description	Acronym	
Country		Factor
Study identification n.	*Study ID*	Covariate
Experiment identification n.	*Exp.ID*	Covariate
Study length	*Study length*	Covariate
Fertilizer type ^(a)^	*Fertilizer type*	Factor
N rate applied	*N rate*	Covariate
Number of fertilizer application	*n. Splits*	Covariate
Mode of fertilizer application ^(b)^	*App. Mode*	Factor
Crop type	*Crop type*	Factor
Crop residues	*Res*	Covariate
Long term precipitation	*Prec*	Covariate
Long term temperature	*Temp*	Covariate
Soil texture	*Soil Texture*	Factor
Soil pH	*pH*	Covariate
Soil tillage	*T*	Factor
Irrigation	*I*	Factor
Soil liming	*L*	Factor
Soil chamber deployment length	*CDL*	Covariate
Soil chamber gas samples	*CGS*	Covariate
		
**(a) Fertilizer Type**	**Acronym**	
Urea	*U*	Factor
Urea with additives	*U & AD*	Factor
Urea with N inhibitor	*U & NI*	Factor
Ammonium nitrate	*AN*	Factor
Ammonium	*A*	Factor
Ammonium with N inhibitor	*A & NI*	Factor
Potassium, Sodium, and Calcium nitrate	*Ni*	Factor
Nitrates with N inhibitor	*Ni & NI*	Factor
Mixture of various synthetic N fertilizers	*Min-N mix*	Factor
Animal manure and other organic fertilizers	*O*	Factor
Organic mix with Mineral-N	*O-Min-N mix*	Factor
Biological N fixing crops	*BNF*	Factor
**(b) Mode of fertilizer application**	**Acronym**	
Surface banding, banding, broadcast	*broadcast*	Factor
Incorporated, solution,	*incorporated*	Factor
Place, banding, sub-surface, banding sub-surface, sub-surface place	*sub-surface banding*	Factor
Sub-surface banding & broadcasted, incorporated & broadcasted, deposition	*mix application*	Factor

**Table 2:** Statistical summary of modelled net-N_2_O-N emissions (Kg N ha^-1^) and annual N_2_O-*EF* (%) across the tropical dataset reported in Table S1, and distinct among continents, countries, crop types, and fertilizer types.

		Net-N_2_O-N				Annual N_2_O-*EF*			
				C.I.				C.I.	
		Mean	Median	Lower	Upper	Mean	Median	Lower	Upper
Continent	Africa	0.8	0.6	0.1	4.5	1.4	1.4	0.3	5.3
	Asia	0.8	0.4	−2.7E-03	24.6	1.1	1.0	0.1	7.8
	Australia	1.3	1.4	5.4E-02	4.3	0.9	0.9	0.2	2.6
	Central & S. America	2.1	0.9	4.0E-05	18.9	1.3	1.2	0.1	6.7
Country	Australia	1.3	1.4	5.4E-02	4.3	0.9	0.9	0.2	2.6
	Brazil	1.4	0.8	4.0E-05	14.9	1.2	1.3	0.1	5.2
	China	1.1	0.9	0.2	4.4	0.9	0.7	0.4	2.4
	Costa Rica	3.9	3.2	0.4	18.9	2.1	1.5	0.4	6.7
	India	0.6	0.4	−2.7E-03	5.8	1.2	1.0	0.1	7.8
	Indonesia	0.7	0.5	7.3E-02	6.1	1.3	1.3	0.2	4.0
	Kenya	0.8	0.6	0.2	2.7	1.4	1.4	0.6	3.1
	Malaysia	11.2	11.2	3.9	24.6	0.7	0.7	0.2	1.5
	Mali	0.6	0.6	0.2	1.4	1.3	1.3	0.4	2.8
	Philippines	0.5	0.5	0.2	1.2	1.0	1.0	0.4	1.9
	Puerto Rico	3.8	2.2	2.4E-02	15.7	0.7	0.8	0.3	1.6
	Thailand	0.2	0.2	6.2E-02	0.5	1.3	1.1	0.4	4.1
	Zimbabwe	0.8	0.8	0.1	4.5	1.5	1.5	0.3	5.3
Crop Type	AC	0.8	0.6	1.6E-02	4.5	1.2	1.1	0.1	7.8
	R	0.6	0.3	−2.7E-03	5.8	na	na	na	na
	PC	2.8	1.5	4.0E-05	24.6	1.2	0.8	0.1	6.7
Fertilizers	AN	2.4	1.3	8.9E-02	18.9	2.1	1.8	0.6	6.7
	Other N Fertilizers	1.1	0.6	5.3E-04	24.6	1.1	1.0	0.2	7.8
	Urea & NI	0.3	0.3	−2.7E-03	2.3	0.7	0.8	0.1	2.9

C.I. corresponds to the 95% confidence interval range. Crop types are classified in *AC* (annual crops including non-flooded rice), *R* (flooded rice), and *PC* (perennial crops). Fertilizer types are grouped in *AN* (Ammonium nitrate), *Urea & NI* (Urea with N inhibitor), and *Other N Fertilizers* (which includes Urea, Urea with additives, Ammonium, Potassium Sodium and Calcium nitrate, mixture of various synthetic N fertilizers, animal manure and other organic fertilizers, Organic mix with Mineral-N). The N_2_O-*EF* in flooded rice crop (*R*) was not calculated as the subpopulation of *R* was characterized by only studies with length shorter than six months.
