# Supplementary material for: Direct Nitrous Oxide Emissions From Tropical And Sub-Tropical Agricultural Systems - A Review And Modelling Of Emission Factors
Source: Sci Rep. 2017 Mar 10;7:44235. doi: 10.1038/srep44235 (PMC5345046; doi:10.1038/srep44235)
Supplement: Supplementary Information [file srep44235-s1.doc]

**Supplementary Information**

1 Summary Statistics

Summary of dataset used in this study, and bivariate correlation between variables in our dataset are given in Table S1 and Fig. S4 and S5.

Table S1: (See excel table attached)

**Statistical modelling methods**

**Model selection**

The approach to determine the best candidate statistical model (model selection) was achieved by initially constructing a core model which included 19 explanatory variables as fixed effects. Restricted maximum likelihood (REML) estimation was used to estimate standard deviations of random effects. The three factors related to the location and identities of the studies (*Country*, *Study ID*, and *Experiment ID*) were handled as nested random effects. Thus, assuming that effects linked to the study-specific characteristics would vary randomly from study to study in the population. In addition, we hypothesized that net-N_2_O-N response in studies with similar soil types (i.e., *Soil type* levels) would also behave in broadly similar ways. Given the number of *Soil type* levels and strong sparsity in the data between *Soil type* levels, we treated *Soil type* as an additive (crossed) random effect, specified using the spline parameterization of random effects in ‘mgcv’, i.e.‘s(*Study length*, bs= “re”)’. Model selection started using a stepwise backwards elimination of the non-significant terms (*p-*values > 0.05). Compared to an automatic selection approach, this permitted to limit the number of models to fit and to get a better understanding of the specific effect of each covariate when added in combination with other terms, while enabling to perform visual checks of the residuals and fitted effects (e.g., smoothers). The difference among treatments, and interaction between factors, was instead explored applying a forward selection procedure. Here the factors with the most significant effects were added one by one in the model in order to understand their significance when added in combination with other terms, and providing the lowest AIC score in the model averaging. On the basis that we did not have *a priori* knowledge on the relationship between the observed net-N_2_O-N emissions and some specific factors, we assumed that the response variable (net-N_2_O-N) could depend on multiple factors in a complicated manner. We thus explored the effect of some parameters in a non-parametric form revealing structure in the data that might otherwise be missed as fixed linear terms (52). The parameter *Study length* was added in the model as non-linear term using a smoothing spline because we expected a priori a non-linear response to study length, although lacking strong theoretical expectation about the shape of the curve.

*Table S2: Summary information of the fitted GAMM model reported in Eq. 3 with estimated parametric effects, associated standard errors, and statistical significance, as well as p-values of non-parametric terms to test whether the smoothed function significantly reduced model deviance. Note that parameters are listed in alphabetical order, and the estimates reported for the categorical parametric terms correspond to the difference between the reference parameter and the respective parameter listed. As annual crops (AC) is the reference level, the estimate of rice (R) reported in the table represents the difference of effect between AC and R. Fertilizer types are reports as AN (Ammonium Nitrate), U&NI (Urea with Nitrification inhibitor), and other N-fertilizer. In the smoothed terms, f_1_ corresponds to the use of thin plate regression spline and ƒ_2_ to an additive random effect. Estimate column shows the effect of the singular terms, and edf is the effective degree of freedom for the model terms (edf with value 1 suggests reduction to a simple linear effect). Finally, R-sq is the deviance explained by the model, Scale est. is the scaled deviance equivalent to the residual sums of squares, and n. the number of observations used to fit the model.*

| Family: Gaussian |  |  |  |  |  |
| --- | --- | --- | --- | --- | --- |
| Formula: | | | | | |
| | Net-N_2_O-N^0.3 ~ *f* _1_ (*Study length*, by=*Crop type*) + *f* _2_ (*Soil type*, “re”) + *Crop type* + *Fertilizer type* : *N rate* + *N rate* | | --- | | | | | | |
| Parametric coefficients: | Estimate | Std. Error | t value | Pr(>|t|) |  |
| (Intercept) | 0.6539806 | 0.0919408 | 7.113 | 2.09e-11 | *** |
| *R* | 0.1775784 | 0.0991241 | 1.791 | 0.0748 | **⋅** |
| *PC* | 0.2078868 | 0.1111939 | 1.870 | 0.0630 | **⋅** |
| *N rate* | 0.0038635 | 0.0005058 | 7.638 | 9.58e-13 | *** |
| *N rate* : *other N-fertilizer* | -0.0022556 | 0.0004031 | -5.595 | 7.36e-08 | *** |
| *N rate* : *Urea & NI* | -0.0037978 | 0.0006358 | -5.973 | 1.08e-08 | *** |
|  |  |  |  |  |  |
| Approximate significance of smooth terms: | edf | Ref.df | F | p-value |  |
| *f* _1_(*Study length* : *AC*) | 1 | 1 | 7.583 | 0.00643 | ** |
| *f* _1_ (*Study length* : *R*) | 1 | 1 | 11.141 | 0.00101 | ** |
| *f* _1_ (*Study length* : *PC*) | 6.38 | 6.38 | 7.493 | 8.95e-07 | *** |
| *f* _2_ (*Soil type*) | 4.077 | 9 | 1.266 | 0.00840 | ** |
|  |  |  |  |  |  |
| R^2^-adj = 0.572 |  |  |  |  |  |
| Scale est. = 0.035603 | n.= 214 |  |  |  |  |
| Significance codes: | <0.001 ‘***’ | 0.001 ‘**’ | 0.01 ‘*’ | 0.05 ‘**⋅**’ | 0.1 ‘ ’ 1 |

**Figure S1:** Diagnostic of modelled annual net-N_2_O-N to the cube root estimated by the GAMM model (Eq.3) and distinct among 8 fertilizer types (a), or between *AN* (Ammonium Nitrate), *U&NI* (Urea with Nitrification inhibitor), and *other N-fertilizer* groups (b). Dashed lines show 95% confidence interval limits. During the model selection the response of net-N_2_O-N emissions across the 8 groups of fertilizer types showed that only Ammonium Nitrate (*AN*) and Urea with Nitrification inhibitor (*U&NI*) treatments were distinguishable from the other 6 fertilizer types (a). On this basis, to improve the predictive power of the model, the parameter *Fertilizer type* was further aggregated into three major groups: *AN*, *other N-fertilizers*, and *U&NI* (b).


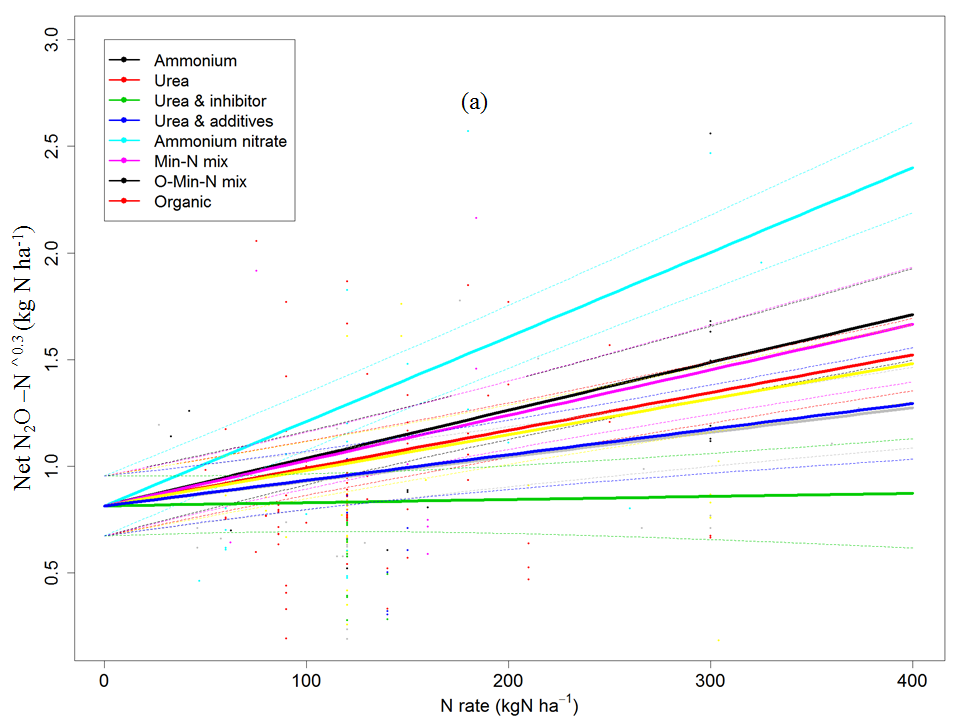


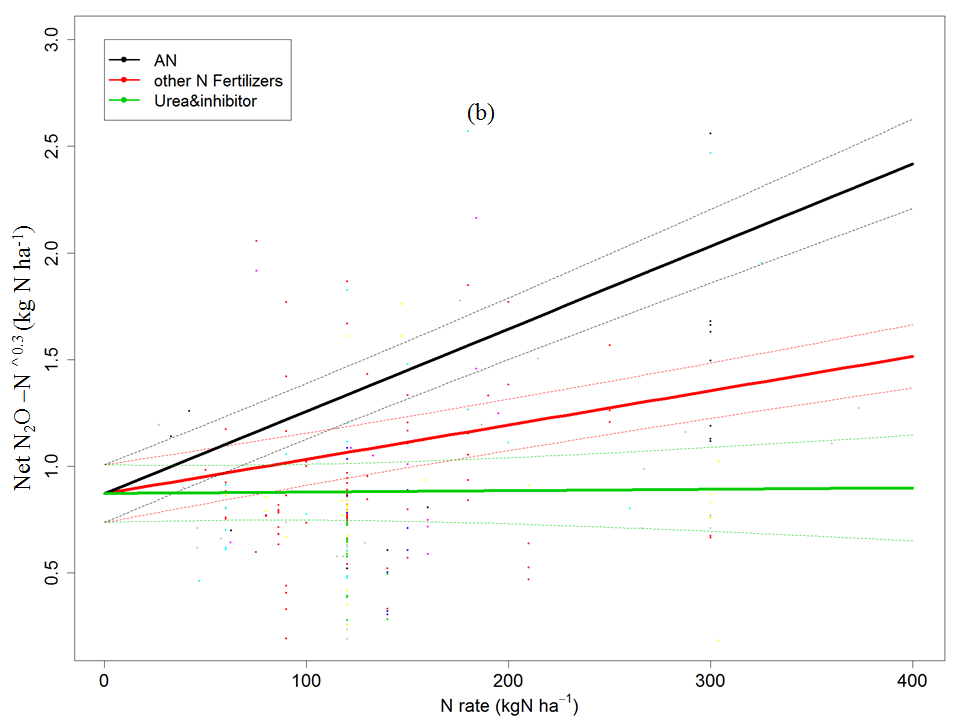


**Figure S2:** Diagnostic of predicted net-N2O emission estimated using the GAMM model (Eq.3). Graph (a) shows the predicted annual net-N2O-N, for *AN* (Ammonium Nitrate), *U&NI* (Urea with Nitrification inhibitor) and *other N-fertilizer* respectively, fixing the terms reported in Eq.3 at: *Study length* = “365 days”, *Soil type* = “Clay”, *N rate* = “from 0 to 250 kg N ha^-1^”, and Country= “India”). Graph (b) shows the time related trends of net-N_2_O estimated assuming the terms reported in Eq.3 at: *Study length* = “from 1 to 365 days”, *Soil type* = “Clay”, *N rate* = “200 kg N ha^-1^”, and Country= “India”). Dashed lines show 95% confidence interval limits. Dashed lines show 95% confidence interval limits. Red lines show the linear regression between modelled net-N_2_O emission (y-axis) and the predictor (x-axis).

**
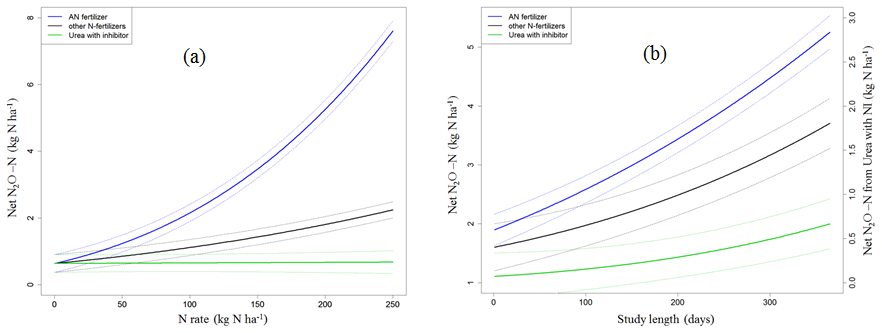
**

**Figure S3:** Fitted effects of the GAMM model (Eq.3) reported in solid lines/curves and corresponding to the smoothed terms *Study length* per *Crop Type*, random effect *Soil Type*, and parametric term *N rate* and *Crop Type* on net-N_2_O-N emissions transformed to the cube root. The 95% confidence interval limits are shown as dashed lines. In the graphs of the smoothed terms *Study lenght* distinct between annual crops (*AC*) (a), rice (*R*) (b), and perennial crops (*PC*) (c), *Soil type* (d). The points shown on the plots are partial residuals given by (ε^partial^= ƒ(*Study L.*) : *Crop type*_i_ + ε) plotted against the factor *Study length*. The ‘rug plots’, along the bottom of each graph, show the locations of the covariate values. While the number in each y-axis caption is the effective degrees of freedom of the term being plotted. In plot (f) the effect of the parameter *AC* is fixed at zero due to *AC* being set as the reference level for crop type.


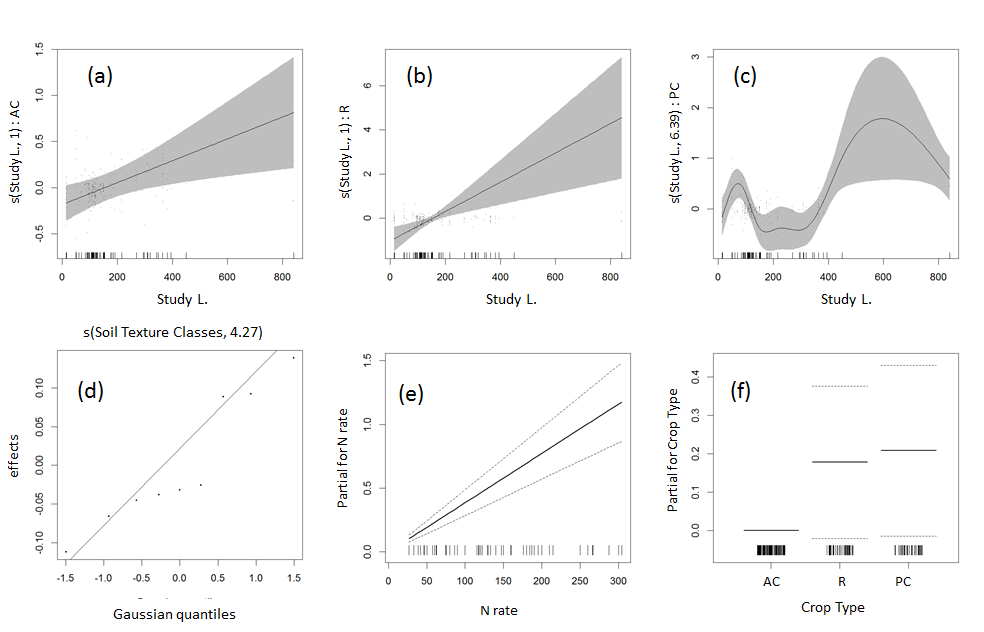


Figure S4: Pie-charts summarizing the missing values across table S.1 and published in peer-review journals on soil N_2_O emission in tropical and sub-tropical croplands. The literature review reported in this study considered as a starting point the literature review reported in Stehfest and Bouwman (28). To report the temporal progresses in the literature, we divided the tropical peer-review publications in the two pie charts that partitioned the studies before (a) and after (b) the year 2006. Both charts report the percentage of missing data among several management practices, soil characteristics, and pre-crop information. In the figure, Lime (soil liming), CT (conventional tillage), Irrig. (irrigation), Res: plant residues, SD (soil drainage), Pre-crop, CEC (cation exchange capacity), BD (soil bulk density).


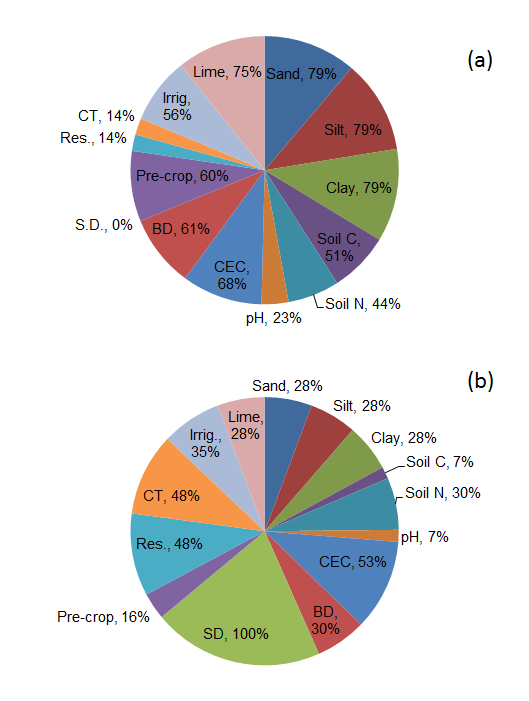


Figure S5: Summary statistics reporting the results of the bivariate analysis among Net-N_2_O-N transformed to the cube root (kg N ha^-1^), length of the study (day), Precipitation (mm), Temperature (˚C), soil pH, number of Splits, and N rate (kg N ha^-1^). Off-Diagonals give bivariate scatterplots of two variables, and lowess regression lines, with black dots indicating observed values.


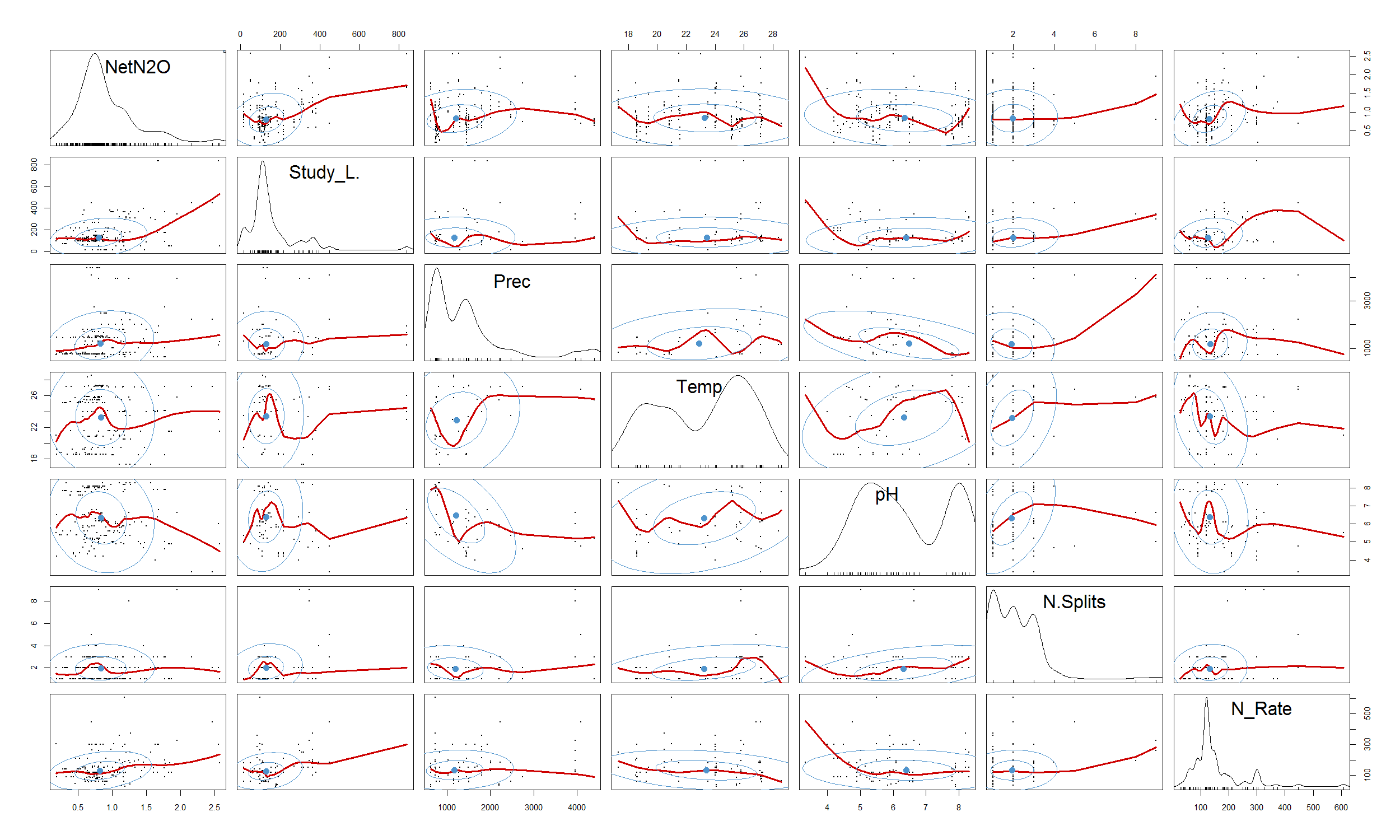


R code for implementing the GAMM model reported in the manuscript. Apply this code with Table S1.2 included as supplementary dataset.

######################################## Start of the R code ###################

#Install and upload packages in R

library(car); library(mgcv);

#Set up the directory where table S1.2 is saved

setwd("C:/ ")

# Use the header of Table S1.2 included in the supplementary information to format an independent dataset, Or to run this code use Table S1.2 where the N2O emissions of the tropical dataset are reported in term of net-N2O emission.

dat<- read.delim("Table_S1.2_Rcode.txt")

-------------------------------------------------------------------

#Reclassify the fertilizer types and remove under-represented classes

dat<- dat[!dat$Fert=="N/A", ]

dat$Fert<- factor(dat$Fert)

dat$FloodRice<- dat$Crop_Type=="Flooded_Rice"

dat$Fertilizers<- as.character(dat$Fert_add)

dat$Fertilizers[dat$Fert_add == "Mineral N mix"]<- "Min-N mix"

dat$Fertilizers[dat$Fert_add == "Mix N Mineral & Organic"]<- "O-Min-N mix"

dat$Fertilizers[dat$Fert_add == "Biological N Fixation"]<- "NfixCrop"

dat$Fertilizers<- factor(dat$Fertilizers, levels= c("Ammonium", "Ammonium & inhibitor", "Urea", "Urea & inhibitor", "Urea & additives", "Ammonium nitrate", "Min-N mix", "Nitrate", "Nitrate & inhibitor", "O-Min-N mix", "Organic", "NfixCrop"))

FertMat<- model.matrix(~Fertilizers-1, dat) # create an indicator variable for each treatment

dat<- cbind(dat, FertMat)

#Remove under-represented fertilizer types

dat2<- dat[!dat$Fertilizers %in% c("Nitrate", "NfixCrop", "Ammonium & inhibitor", "Nitrate & inhibitor"), ]

dat2$Fertilizers<- factor(dat2$Fertilizers)

#Remove Bare soil

dat3<- dat2[!dat2$Crop_Type %in% c("Bare soil"), ]

dat3$Crop_Type<- factor(dat3$Crop_Type)

# Make a new classification for the fertilizers distinct into 3 classes

dat3$Fert.<- as.character (dat3$Fertilizers)

dat3$Fert.[dat3$Fert. == "Ammonium nitrate"] <- "AN"

dat3$Fert.[dat3$Fert. == "Urea & inhibitor"] <- "Urea&inhibitor"

dat3$Fert.[dat3$Fert. %in% c("Min-N mix", "Ammonium", "Organic", "O-Min-N mix", "Urea & additives", "Urea")] <- "other N Fertilizers"

dat3$Fert.<- factor(dat3$Fert.)

# Run the GAMM model reported in Eq. 3. using 8 classes of fertilizer type (see Figure S1a)

lmc<-lmeControl(niterEM=2000, msMaxIter=2000)

GAMM_Model<- gamm(Net_N2O^0.3 ~ s(Study_L., by=Crop_Type) + Crop_Type + Fertilizers:N_Rate + N_Rate + s(USDAtxt, bs="re"),

random=list(Country=~1, Flow=~1, RefN=~1), family= gaussian, data= dat3, subset= N_Rate < 320, method= "REML",control=lmc)

#Plot the prediction results of above GAMM Model

mar.default <- c(4,1,1,1) + 0.1

par(mar = mar.default + c(0, 4, 0, 0))

plot(dat3$Net_N2O^0.3 ~ dat3$N_Rate, col= dat3$Fertilizers, xlim=c(1,400), ylim=c(0.2, 3), pch=20, cex=0.5, xlab = substitute(paste("N rate (kgN " * "ha"^{-1} * ")", list(x=""))),

ylab = expression("net-N"[2] * "O"^{"^"}^{0.3} * " " * "(kgN"[2] * "O" * " " * "ha"^{-1} * ")"), cex.lab=1.5, cex.axis=1.5)

NRseq<- seq(0, 400, l= 50)

colvec<- 1:9; names(colvec)<- levels(dat3$Fertilizers)

for(i in unique(dat3$Fertilizers)){

fitted_Net_N2O<- predict(GAMM_Model$gam, newdata= data.frame(Study_L.=365, USDAtxt= "Clay", Crop_Type= "Annual non flooded crops", Fertilizers= i, N_Rate= NRseq, Country= "India", Fllow=1), se.fit= T)

lines(fitted_Net_N2O$fit ~ NRseq, col= colvec[i], lwd= 4)

lines(fitted_Net_N2O$fit + fitted_Net_N2O$se.fit ~ NRseq, col= colvec[i], lwd= 1, lty= 2)

lines(fitted_Net_N2O$fit - fitted_Net_N2O$se.fit ~ NRseq, col= colvec[i], lwd= 1, lty= 2)

}

legend(x= 0, y = 3, legend = levels(dat3$Fertilizers), col = 1:6, lty= 1, lwd= 2, pch=20, cex=1.4)

# Run the GAMM model reported in Eq. 3. using only the 3 broad classes of fertilizer type (see Figure S1b)

GAMM_Model_2<- gamm(Net_N2O^0.3 ~ s(Study_L., by=Crop_Type) + Crop_Type + Fert.:N_Rate + N_Rate + s(USDAtxt, bs="re"),

random=list(Country=~1, Flow=~1, RefN=~1), family= gaussian, data= dat3, subset= N_Rate < 320, method= "REML",control=lmc)

#Plot the prediction results of above GAMM Model2

mar.default <- c(4,1,1,1) + 0.1

par(mar = mar.default + c(0, 4, 0, 0))

plot(dat3$Net_N2O^0.3 ~ dat3$N_Rate, col= dat3$Fertilizers, xlim=c(1,400), ylim=c(0.2, 3), pch=20, cex=0.5, xlab = substitute(paste("N rate (kgN " * "ha"^{-1} * ")", list(x=""))),

ylab = expression("net-N"[2] * "O"^{"^"}^{0.3} * " " * "(kgN"[2] * "O" * " " * "ha"^{-1} * ")"), cex.lab=1.5, cex.axis=1.5)

NRseq<- seq(0, 400, l= 50)

colvec<- 1:9; names(colvec)<- levels(dat3$Fert.)

for(i in unique(dat3$Fert.)){

fitted_Net_N2O<- predict(GAMM_Model_2$gam, newdata= data.frame(Study_L.=365, USDAtxt= "Clay", Crop_Type= "Annual non flooded crops", Fert.= i, N_Rate= NRseq, Country= "India"), se.fit= T)

lines(fitted_Net_N2O$fit ~ NRseq, col= colvec[i], lwd= 4)

lines(fitted_Net_N2O$fit + fitted_Net_N2O$se.fit ~ NRseq, col= colvec[i], lwd= 1, lty= 2)

lines(fitted_Net_N2O$fit - fitted_Net_N2O$se.fit ~ NRseq, col= colvec[i], lwd= 1, lty= 2)

}

legend(x= 0, y = 3, legend = levels(dat3$Fert.), col = 1:6, lty= 1, lwd= 2, pch=20, cex=1.4)

#Diagnostic plots of GAMM Model 2

type <- "deviance" ## "pearson" & "response" are other valid choices

resid <- residuals(GAMM_Model_2$gam, type = type)

linpred <- napredict(GAMM_Model_2$gam$na.action, GAMM_Model_2$gam$linear.predictors)

observed.y <- napredict(GAMM_Model_2$gam$na.action, GAMM_Model_2$gam$y)

#Note the last two lines are applying the NA handling method used when the model was fitted to the information on the linear.predictors

#and y, the stored copy of the response data.

# Reproduce Figure 3a of the manuscript

qq.gam(GAMM_Model_2$gam, rep = 0, level = 0.9, type = type, rl.col = 2,

rep.col = "gray80")

#Figure 3c

hist(resid, xlab = "Residuals", main = "Histogram of residuals")

#Figure 3b

plot(linpred, resid, main = "Resids vs. linear pred.",

xlab = "linear predictor", ylab = "residuals")

abline(0,0, col= "black")

#Figure 3d

plot(fitted(GAMM_Model_2$gam), observed.y, xlab = "Fitted Values",

ylab = "Response", main = "Response vs. Fitted Values")

abline(lm(observed.y~fitted(GAMM_Model_2$gam)), lwd=2, col= "black")

abline(0,1, lwd=2, col= 8)

legend("bottomright", legend=c("predicted ~ observed", "1:1"), col=c(1,8), lty=1, lwd=2)

#add regression

fit <- lm(observed.y~fitted(GAMM_Model_2$gam))

lgd <- c(

paste("R^2 =", round(summary(fit)$r.squared,3)),

paste("Slope =", round(coef(fit)[2],3))

)

legend("topleft", legend=lgd)

abline(fit, lwd=2)

# Reproduce Figure S2a of the Supplementary Information on the effect of Fertilizers and N rate on annual net-N2O emissions (see Figure S2a)

fitted_AN<- predict(GAMM_Model_2$gam, newdata= data.frame(Study_L.=365, USDAtxt= "Clay", Crop_Type= "Annual non flooded crops", Fert.= "AN", N_Rate= 0:250, n.Splits= "one", Country= "India", Fllow= 1, RefN= 45), se.fit= T)

plot(x= 0:250, y= fitted_AN$fit^3.3333333333333335, type= "l")

x<- 0:250

y1<- fitted_AN$fit^3.3333333333333335

abline(lm(y1~x), col= "red")

fitted_otherNFert<- predict(GAMM_Model_2$gam, newdata= data.frame(Study_L.=365, USDAtxt= "Clay", Fert.= "other N Fertilizers", Crop_Type= "Annual non flooded crops", N_Rate= 0:250, Country= "India", Fllow= 1, RefN= 45), se.fit= T)

plot(x= 0:250, y= fitted_otherNFert$fit^3.3333333333333335, type= "l")

x<- 0:250

y2<- fitted_otherNFert$fit^3.3333333333333335

abline(lm(y2~x), col= "red")

fitted_Ureainhibitor<- predict(GAMM_Model_2$gam, newdata= data.frame(Study_L.=365, USDAtxt= "Clay", Fert.= "Urea&inhibitor", Crop_Type= "Annual non flooded crops", N_Rate= 0:250, Country= "India", Fllow= 1, RefN= 45), se.fit= T)

plot(x= 0:250, y= fitted_Ureainhibitor$fit^3.3333333333333335, type= "l")

x<- 0:250

y3<- fitted_Ureainhibitor$fit^3.3333333333333335

abline(lm(y3~x), col= "red")

par(mar=c(5,6,1,5)+.1)

plot(x,y1, type="l",col=4, ylab = expression("Net-N"[2] * "O" * " " * "(kgN"[2] * "O" * " " * "ha"^{-1} * "yr"^{-1} * ")"),

xlab=substitute(paste("N rate (kgN " * "ha"^{-1} * ")", list(x=""))), ylim=c(0, 8), cex.lab=1.5, cex.axis=1.5)

lines(x,y1,col=4,lwd=3)

lines(x, y1+1.96*fitted_AN$se.fit, col= 4, lwd= 1, lty= 2)

lines(x, y1-1.96*fitted_AN$se.fit, col= 4, lwd= 1, lty= 2)

par(new=TRUE)

plot(x,y2,,type="l",col=1, ylab = expression("Net-N"[2] * "O" * " " * "(kgN"[2] * "O" * " " * "ha"^{-1} * "yr"^{-1} * ")"),

xlab=substitute(paste("N rate (kgN " * "ha"^{-1} * ")", list(x=""))), ylim=c(0, 8), cex.lab=1.5, cex.axis=1.5)

lines(x,y2,col=1,lwd=3)

lines(x, y2+1.96*fitted_otherNFert$se.fit, col= 1, lwd= 1, lty= 2)

lines(x, y2-1.96*fitted_otherNFert$se.fit, col= 1, lwd= 1, lty= 2)

par(new=TRUE)

plot(x,y3,type="l",col=3, ylab = expression("Net-N"[2] * "O" * " " * "(kgN"[2] * "O" * " " * "ha"^{-1} * "yr"^{-1} * ")"),

xlab=substitute(paste("N rate (kgN " * "ha"^{-1} * ")", list(x=""))), ylim=c(0, 8), cex.lab=1.5, cex.axis=1.5)

lines(x,y3,col=3,lwd=3)

lines(x, y3+1.96*fitted_Ureainhibitor$se.fit, col= 3, lwd= 1, lty= 2)

lines(x, y3-1.96*fitted_Ureainhibitor$se.fit, col= 3, lwd= 1, lty= 2)

legend("topleft",col=c("4","1", "3"),lty=1,legend=c("AN fertilizer","other N-fertilizers", "Urea with inhibitor"),lwd= 2, cex=1.4)

# Reproduce Figure S2b of the Supplementary Information on the effect of effect of time and N rate on net-N2O net-N2O emissions (see Figure S2b)

fitted_StudyL_AN<- predict(GAMM_Model_2$gam, newdata= data.frame(Study_L.=1:365, USDAtxt= "Clay", Crop_Type= "Annual non flooded crops", Fert.= "AN", N_Rate= 200, Country= "India", Fllow= 1, RefN= 45), se.fit= T)

plot(x= 1:365, y= fitted_StudyL_AN$fit^3.3333333333333335, type= "l")

x<- 1:365

y1<- fitted_StudyL_AN$fit^3.3333333333333335

abline(lm(y1~x), col= "red")

fitted_StudyL__otherNFert<- predict(GAMM_Model_2$gam, newdata= data.frame(Study_L.=1:365, USDAtxt= "Clay", Fert.= "other N Fertilizers", Crop_Type= "Annual non flooded crops", N_Rate= 200, Country= "India", Fllow= 1, RefN= 45), se.fit= T)

plot(x= 1:365, y= fitted_StudyL__otherNFert$fit^3.3333333333333335, type= "l")

x<- 1:365

y2<- fitted_StudyL__otherNFert$fit^3.3333333333333335

abline(lm(y2~x), col= "red")

fitted_StudyL_Ureainhibitor<- predict(GAMM_Model_2$gam, newdata= data.frame(Study_L.=1:365, USDAtxt= "Clay", Fert.= "Urea&inhibitor", Crop_Type= "Annual non flooded crops", N_Rate= 200, Country= "India", Fllow= 1, RefN= 45), se.fit= T)

plot(x= 1:365, y= fitted_StudyL_Ureainhibitor$fit^3.3333333333333335, type= "l")

x<- 1:365

y3<- fitted_StudyL_Ureainhibitor$fit^3.3333333333333335

abline(lm(y3~x), col= "red")

par(mar=c(5,6,1,5)+.1)

plot(x,y1, type="l",col=4, ylab = expression("Net-N"[2] * "O" * " " * "(kgN"[2] * "O" * " " * "ha"^{-1} * ")"), xlab=" Study lenght (days)", ylim=c(1, 5.5), cex.lab=1.5, cex.axis=1.5)

lines(x,y1,col=4,lwd=3)

lines(x, y1+1.96*fitted_StudyL_AN$se.fit, col= 4, lwd= 1, lty= 2)

lines(x, y1-1.96*fitted_StudyL_AN$se.fit, col= 4, lwd= 1, lty= 2)

par(new=TRUE)

plot(x,y2,type="l",col=1, xaxt="n",yaxt="n", ylab = expression("Net-N"[2] * "O" * " " * "(kgN"[2] * "O" * " " * "ha"^{-1} * ")"), xlab=" Study lenght (days)", ylim=c(0, 3), cex.lab=1.5, cex.axis=1.5)

lines(x,y2,col=1,lwd=3)

lines(x, y2+1.96*fitted_StudyL_AN$se.fit, col= 1, lwd= 1, lty= 2)

lines(x, y2-1.96*fitted_StudyL_AN$se.fit, col= 1, lwd= 1, lty= 2)

par(new=TRUE)

plot(x,y3,,type="l",col=3, xaxt="n",yaxt="n", ylab = expression("Net-N"[2] * "O" * " " * "(kgN"[2] * "O" * " " * "ha"^{-1} * ")"), xlab=" Study lenght (days)", ylim=c(0, 3), cex.lab=1.5, cex.axis=1.5)

lines(x,y3,col=3,lwd=3)

lines(x, y3+1.96*fitted_StudyL_AN$se.fit, col= 3, lwd= 1, lty= 2)

lines(x, y3-1.96*fitted_StudyL_AN$se.fit, col= 3, lwd= 1, lty= 2)

axis(4, cex.axis=1.5)

mtext(expression("net-N"[2] * "O" * " " * "from" * " " * "Urea" * " " * "with" * " " * "NI" * " " * "(kgN"[2] * "O" * " " * "ha"^{-1} * ")"), xlab=" Study lenght (days)",side=4,line=3, cex=1.5)

legend("topleft",col=c("4","1", "3"),lty=1,legend=c("AN fertilizer","other N-fertilizers","Urea with inhibitor"),lwd= 2, cex=1.4)

#Extract the intercept and the coefficients of the GAMM Model2.

coef(GAMM_Model_2$gam)

######################################## End of the R code ###################
